# Comparative Cardiovascular Outcomes of GLP‐1 Receptor Agonists Versus Bariatric Surgery: A Systematic Review and Network Meta‐Analysis

**DOI:** 10.1002/edm2.70311

**Published:** 2026-09-05

**Authors:** Erfan Shirmohammadi, Negar Ghasemloo, Navid Ebrahimi, Narjes Mohammadzadeh, Amir Ehtemami, Mahan Babaei, Shakiba Fardoost

**Affiliations:** ^1^ Department of Surgery, Imam Khomeini Hospital Tehran University of Medical Sciences Tehran Iran; ^2^ School of Medicine Tehran University of Medical Sciences Tehran Iran; ^3^ School of Medicine Shahid Beheshti University of Medical Sciences Tehran Iran; ^4^ Iran Azad University of Medical Sciences, Tehran Medical Branch Tehran Iran

**Keywords:** bariatric surgery, cardiovascular diseases, cardiovascular risk reduction, GLP‐1 receptor agonists, heart failure, major adverse cardiovascular events (MACE), myocardial infarction, network meta‐analysis, obesity, systematic review

## Abstract

**Background:**

Obesity, a major global health issue, heightens the risk of cardiovascular diseases, including major adverse cardiovascular events (MACE), heart failure (HF) and myocardial infarction (MI). This study provides the first network meta‐analysis (NMA) integrating direct and indirect evidence to compare the cardiovascular efficacy of bariatric surgery (BS) and glucagon‐like peptide‐1 receptor agonists (GLP‐1RAs) in individuals with obesity.

**Methods:**

A systematic review and NMA were performed in adherence to PRISMA guidelines. PubMed, Embase, Cochrane Library and Google Scholar were searched for studies published from January 2000 to September 2024. Eligible studies reported cardiovascular outcomes, specifically MACEs, HF and MI, in patients with obesity receiving BS, GLP‐1RAs or control treatment. Fixed‐ and random‐effects models were used to analyse hazard ratios (HRs) and risk ratios (RRs), with heterogeneity assessed with *I* ^2^ and consistency through node‐splitting analysis.

**Results:**

Forty‐three studies involving 932,380 patients were included BS was associated with significantly greater reductions in MACEs (HR 0.66; 95% CI 0.60–0.74), HF (HR 0.45; 95% CI 0.38–0.53) and MI (HR 0.53; 95% CI 0.45–0.63) compared with controls in random‐effects models. GLP‐1RAs reduced MACEs (HR 0.85; 95% CI 0.77–0.94) but showed non‐significant effects on HF and MI. Despite high heterogeneity (*I* ^2^: 66%–93%), directional consistency was observed across studies.

**Conclusions:**

BS demonstrated more consistent and statistically significant reductions in all cardiovascular outcomes compared to GLP‐1RAs, which showed more variable efficacy, particularly for HF and MI. Nevertheless, GLP‐1RAs remain effective evidence‐based alternatives for cardiovascular risk reduction in patients who are not candidates for surgery, but future studies on newer GLP‐1RAs are required. The high heterogeneity across studies highlights the need for cautious interpretation. These results may inform individualized treatment selection in patients with obesity at high cardiovascular risk.

## Graphical abstract

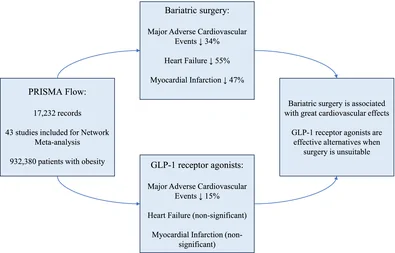

**Abbreviations**
BS: bariatric surgery
CV: cardiovascular
GLP‐1RAs: glucagon‐like peptide‐1 receptor agonists
HF: heart failure
HRs: hazard ratios
MACEs: major adverse cardiovascular events
MI: myocardial infarction
NMA: network meta‐analysis
PRISMA: Preferred Reporting Items for Systematic Reviews and Meta‐Analyses
RCTs: randomized controlled trials
RoR: ratio of odds ratios
RRs: risk ratios

## 1 Introduction

The World Obesity Atlas 2023 reports that 38% of the global population is currently overweight or obese, projected to rise to 51% by 2035 [1]. The WHO reports that adult and adolescent obesity rates have doubled and quadrupled respectively since 1990 [2].

Obesity exacerbates cardiovascular (CV) risk factors like dyslipidemia, hypertension, sleep apnea and prothrombotic state [3, 4]. Obesity significantly increases the risk of heart failure (HF), myocardial infarction (MI) and major adverse cardiovascular events (MACEs) [5, 6]. A UK Biobank study shows individuals with obesity and diabetes have a 96% higher CV mortality risk than those with normal weight and no diabetes [7]. Obesity and diabetes drive the rise in HF, with obesity and intermediate hypertension increasing lifetime HF risk by 24%–62% [8]. Furthermore, individuals with obesity are associated with an approximately 10% increase in developing MI compared to those with normal weight [9].

Among obesity management strategies, pharmacological and surgical interventions have the most substantial impact on reducing CV complications [10, 11, 12]. Glucagon‐like peptide‐1 receptor agonists (GLP‐1RAs), namely liraglutide and semaglutide, effectively reduce CV risk in obesity [13]: MACEs by 14%, HF by 11%, MI by 10%, stroke by 17% and CV mortality by 13% [14]. On the other hand, BS also significantly reduces CV risks in obesity with a 50% decrease in HF, 42% in MI, 36% in stroke and 41% in CV mortality [15].

Although evidence supports the CV benefits of both BS and GLP‐1RAs in individuals with obesity, only a few direct comparisons between these interventions are available [16, 17]. This limited head‐to‐head evidence makes it challenging to determine which approach offers greater efficacy for reducing CV events. To the best of our knowledge, we conducted the first network meta‐analysis (NMA) integrating both direct and indirect comparisons (in the absence of large‐scale head‐to‐head trials) to evaluate the effects of BS and GLP‐1RAs on CV events. This analysis aims to inform clinical decision‐making and personalizing treatments for diminishing CV risks associated with obesity.

## 2 Methods

### 2.1 Study Design

The present systematic review and NMA compares the effects of BS and GLP‐1RAs on cardiovascular outcomes in individuals with obesity. The network geometry was characterized by three nodes (BS, GLP‐1RAs and control) connected by evidence from included studies, with both direct and indirect comparisons incorporated into the network model. The transitivity assumption (a prerequisite for valid indirect comparisons in NMA) was evaluated by examining the distribution of potential effect modifiers, including study design, follow‐up duration, baseline patient characteristics and intervention type, across the treatment comparisons. We considered the assumption to be plausible given the broadly similar patient populations and outcome definitions across included studies.

This study was reported in accordance with the Preferred Reporting Items for Systematic Reviews and Meta‐Analyses (PRISMA) guidelines [18]. Although prospective registration in PROSPERO was not completed, this review followed a pre‐specified internal protocol and adhered strictly to PRISMA methodology to minimize potential reporting bias.

### 2.2 Search Strategy

Literature search was carried out using PubMed/MEDLINE, Ovid/Embase, Google Scholar and Cochrane Library. The search strategy was developed using a combination of Medical Subject Headings (MeSH) and additional relevant terms to ensure comprehensive coverage of the topic. Keywords included: ‘Bariatric Surgery’, ‘Metabolic Surgery’, ‘Glucagon‐Like Peptide‐1 Receptor Agonists’, ‘GLP‐1 agonist’ and ‘GLP‐1RA’ combined with ‘Cardiovascular disease’, ‘cardiovascular risk factors’, ‘Major Adverse Cardiovascular Event’ and ‘MACE’. Citations of selected articles and any relevant studies that evaluated GLP‐1RAs or bariatric/metabolic surgery with CV outcomes were reviewed. All publications from January 2000 to September 30, 2024 were evaluated.

### 2.3 Eligibility Criteria

Studies were included if they met the following criteria: (1) The primary focus was on comparing CV outcomes, particularly MACEs, among individuals with obesity across three groups, including those undergoing BS versus control, GLP‐1RAs versus control and BS versus GLP‐1RAs treatments; (2) the primary outcomes included MACEs, defined as all‐cause mortality or the first occurrence of MI, stroke, HF hospitalization, or revascularization events; (3) the study population consisted of adults aged 18–80 years with obesity and associated CV risk factors (e.g., type 2 diabetes, hypertension, dyslipidemia), excluding individuals with pregnancy or active malignancy; (4) only studies with accessible full‐texts were considered; (5) study designs included randomized controlled trials (RCTs) or observational cohort studies; and (6) articles were published in peer‐reviewed journals and available in English. Exclusion criteria encompassed case reports, reviews, editorials and studies focusing solely on interventions not involving BS or GLP‐1RAs.

### 2.4 Study Selection and Data Collection and Extraction

**FIGURE 1:**
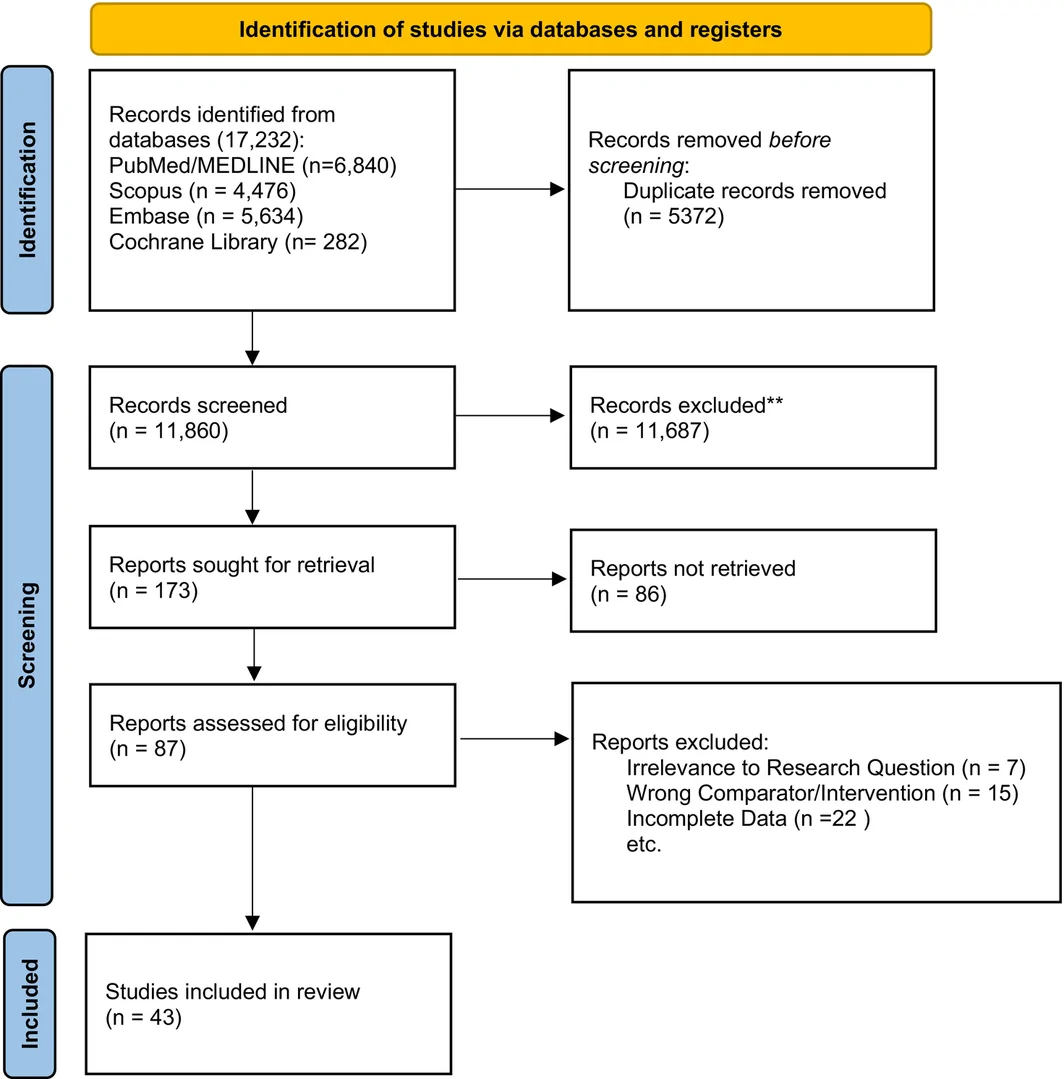
The PRISMA flow diagram of the literature search.

The literature review process was conducted by two independent reviewers. In cases of disagreement or lack of concordance during the study selection evaluation, resolutions were achieved through discussions involving additional investigators to reach a consensus. The screening involved evaluating the relevance of study titles and abstracts and duplicate entries were identified and removed using Endnote Reference Manager. A total of 87 articles were reviewed in detail, with 43 studies ultimately included in the analysis. Detailed information regarding the included studies is provided in Table 1. Details regarding the exclusion of studies are provided in the Preferred Reporting Items for PRISMA flowchart (Figure 1). Two reviewers independently conducted data extraction using a standardized form to maintain consistency and accuracy. The extracted information included key study characteristics, such as the author, year of publication and study design, as well as patient demographics and relevant details regarding BS or GLP‐1RA and CV outcomes.

**TABLE 1:** Characteristics of the included studies.

REF	Author name (year)	Study design	Study groups	No. of cases	No. of controls	Risk of bias^1^
[19]	Bouchard et al. 2022	Cohort	Bariatric vs. Cont	3627	5420	Good quality
[20]	Alkharaiji et al. 2019	Cohort	Bariatric vs. Cont	131	579	Good quality
[21]	Sampalis et al. 2006	Cohort	Bariatric vs. Cont	1035	5746	Good quality
[22]	Romeo et al. 2012	Cohort	Bariatric vs. Cont	345	262	Good quality
[23]	Douglas et al. 2015	Cohort	Bariatric vs. Cont	3882	3882	Good quality
[24]	Eliasson et al. 2015	Cohort	Bariatric vs. Cont	6132	6132	Good quality
[25]	Benotti et al. 2017	Cohort	Bariatric vs. Cont	1724	1724	Good quality
[26]	Brown et al. 2022	Cohort	Bariatric vs. Cont	60,445	268,362	Good quality
[27]	Moussa et al. 2020	Cohort	Bariatric vs. Cont	3701	3701	Good quality
[28]	Stenberg et al. 2020 (I)	Cohort	Bariatric vs. Cont	11,863	26,199	Good quality
[29]	Wong et al. 2021	Cohort	Bariatric vs. Cont	303	1399	Good quality
[30]	Hoskuldsdottir et al. 2020	Cohort	Bariatric vs. Cont	387	387	Good quality
[31]	Dash et al. 2021	Cohort	Bariatric vs. Cont	3098	5470	Good quality
[32]	Hung et al. 2021	Cohort	Bariatric vs. Cont	1436	1436	Good quality
[33]	Lundberg et al. 2021	Cohort	Bariatric vs. Cont	28,204	40,827	Good quality
[34]	Yuan et al. 2021	Cohort	Bariatric vs. Cont	308	701	Good quality
[35]	Mentias et al. 2022	Cohort	Bariatric vs. Cont	94,885	94,885	Good quality
[36]	Aminian et al. 2019	Cohort	Bariatric vs. Cont	2287	39,267	Good quality
[37]	Singh et al. 2020	Cohort	Bariatric vs. Cont	5170	9995	Good quality
[38]	Rassen et al. 2021	Cohort	Bariatric vs. Cont	344	551	Good quality
[39]	Persson et al. 2017	Cohort	Bariatric vs. Cont	22,295	25,564	Good quality
[40]	Jamaly et al. 2019	Cohort	Bariatric vs. Cont	2003	2030	Good quality
[41]	Liakopoulos et al. 2020	Cohort	Bariatric vs. Cont	5321	5321	Good quality
[42]	Hoskuldsdottir et al. 2021	Cohort	Bariatric vs. Cont	5321	5321	Good quality
[43]	Doumouras et al. 2021	Cohort	Bariatric vs. Cont	1319	1319	Good quality
[44]	Naslund et al. 2021	Cohort	Bariatric vs. Cont	509	509	Good quality
[45]	Pirlet et al. 2020	Cohort	Bariatric vs. Cont	116	116	Good quality
[46]	Sjostrom, et al. 2012	Cohort	Bariatric vs. Cont	2010	2037	Good quality
[47]	Kosiborod et al. 2024 (I)	RCT	Glp1 vs. Cont	310	306	Low risk
[48]	Kosiborod et al. 2023 (II)	RCT	Glp1 vs. Cont	263	266	Low risk
[49]	Lincoff et al. 2023	RCT	Glp1 vs. Cont	8801	8803	Low risk
[50]	Ruff et al. 2022	RCT	Glp1 vs. Cont	2075	2081	Low to moderate risk
[51]	Gerstein et al. 2021 (I)	RCT	Glp1 vs. Cont	2717	1359	Low risk
[52]	Marso et al. 2020 (I)	RCT	Glp1 vs. Cont	4668	4672	Low risk
[53]	Gerstein et al. 2019 (II)	RCT	Glp1 vs. Cont	4949	4952	Low risk
[54]	Husain et al. 2019	RCT	Glp1 vs. Cont	1591	1592	Low risk
[55]	Hernandez et al. 2018	RCT	Glp1 vs. Cont	4731	4732	Low risk
[56]	Margulies et al. 2016	RCT	Glp1 vs. Cont	154	146	Low risk
[57]	Holman et al. 2017	RCT	Glp1 vs. Cont	7356	7396	Low risk
[58]	Marso et al. 2016 (II)	RCT	Glp1 vs. Cont	1648	1649	Low risk
[59]	Pfeffer et al. 2015	RCT	Glp1 vs. Cont	3034	3034	Low risk
[16]	Dicker et al. 2024	Cohort	Glp1 vs. Bariatric	2371	2371	Good quality
[17]	Stenberg et al. 2024 (II)	Cohort	Glp1 vs. Bariatric	2039	2039	Good quality
[60]	Stenberg et al. 2023 (III)	Cohort	Glp1 vs. Bariatric	2161	2161	Good quality
[61]	Wolff Sagy et al. 2024	Cohort	Glp1 vs. Bariatric	2205	2205	Good quality

^1^ Risk of bias assessment was performed using the Newcastle‐Ottawa Scale (NOS) for cohort studies, while the Cochrane Risk of Bias Tool I (RoB I) was used for RCTs.

The risk of bias was independently assessed by two authors using the Cochrane Risk of Bias Tool (RoB I) for RCTs and the Newcastle‐Ottawa Scale (NOS) for observational studies that met the inclusion criteria. All the included RCT studies reported a central randomization process, objectively determined outcomes and all primary and secondary outcomes as pre‐specified in their protocols; therefore the risk of bias for selective reporting was judged as low across most domains; detailed domain‐level assessments are provided individually in the Supporting Information.

### 2.5 Statistical Analysis

The NMA was conducted using R software. Both fixed‐effects and random‐effects models were used to account for variability between studies. Heterogeneity was assessed using the *I* ^2^ statistic. Inconsistency between direct and indirect comparisons was evaluated through node‐splitting analysis using both common effects and random effects models, in which the direct and indirect evidence for each comparison are separated and compared to ensure the reliability of the network model. A statistically significant difference between direct and indirect estimates (*p* < 0.05) was used as the threshold for identifying potential inconsistency. Potential sources of heterogeneity were narratively explored in the discussion.

Hazard ratios (HRs) were extracted along with the number of events (e.g., MACEs, HF, MI) and total patient numbers. The lack of HRs in a subset of studies necessitated the calculation of risk ratios (RRs) as a complementary measure to maximize data utilization and minimize exclusion of eligible evidence. HR‐ and RR‐based NMA were conducted separately and are reported in parallel (i.e., effect measures were not pooled across analysis types). While effect measures were not pooled across analysis types, the consistency of findings between HR and RR analyses serves as an internal validation, suggesting that the observed trends are robust across different statistical reporting methods. Sensitivity analyses were conducted by excluding studies with a higher risk of bias and those with fewer than 1000 participants. As these exclusions did not lead to significant changes in results, they are not included in the results section. These approaches ensured a robust and consistent analysis while addressing potential sources of bias.

## 3 Results

### 3.1 Study Characteristics

A total of 932,380 patients were included across all comparisons. The study groups consisted of 830,215 patients in the BS versus control comparison, 83,285 patients in the GLP‐1RAs versus control comparison and 18,880 patients in the BS versus GLP‐1RAs comparison. The treatment groups comprised 321,374 patients, while the control groups included 611,006 patients (Table S1).

### 3.2 Comparison of MACEs

**FIGURE 2:**
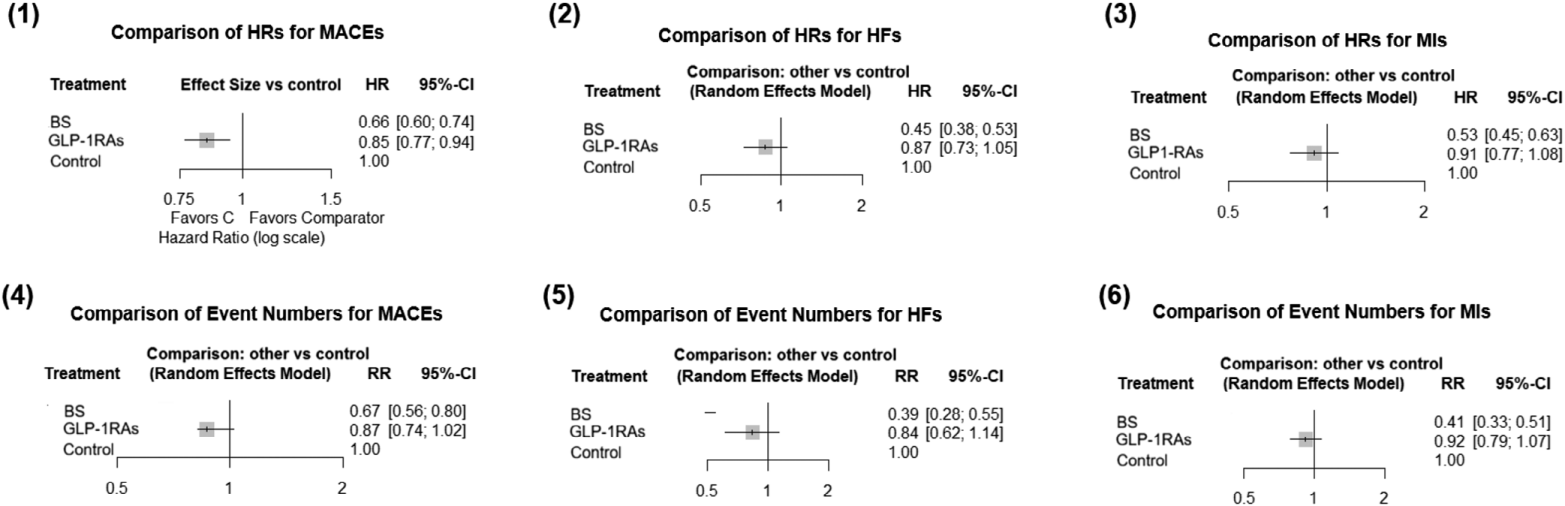
Network meta‐analysis forest plots for HRs and RRs across Bariatric surgeries (BS), Glucagon‐like peptide‐1 receptor agonists (GLP‐1RAs) and control. Estimates are presented with 95% confidence intervals (95% CI) under random‐effects models. Panels 2.1, 2.4: Major adverse cardiovascular events (MACEs). Panels 2.2, 2.5: Heart failure (HF) events. Panels 2.3, 2.6: Myocardial infarction (MI).

BS was associated with a significant reduction in MACEs compared with controls in both fixed‐ and random‐effects models. A total of 25 studies provided data on HRs for MACEs, with 25 pairwise comparisons across BS, GLP‐1RAs and control. In the fixed‐effects model, BS versus control yielded an HR of 0.73 (95% CI: 0.70–0.76; *Z* = −14.59; *p* < 0.001), while GLP‐1RAs versus control showed an HR of 0.87 (95% CI: 0.84–0.91; *Z* = −6.06; *p* < 0.001). In the random‐effects model, BS versus control showed an HR of 0.66 (95% CI: 0.60–0.74; *Z* = −7.67; *p* < 0.001) GLP‐1RAs versus control had an HR of 0.85 (95% CI: 0.77–0.94; *Z* = −3.10; *p* = 0.002) (Figure 2, panel 2.1).

For RRs, 23 studies provided data on the number of MACEs, with 23 pairwise comparisons. In the fixed‐effects model, BS versus control yielded an RR of 0.78 (95% CI: 0.74–0.82; *Z* = −10.31; *p* < 0.001), while GLP‐1RAs versus control had an RR of 0.89 (95% CI: 0.86–0.93; *Z* = −5.58; *p* < 0.001). In the random‐effects model, BS versus control showed an RR of 0.67 (95% CI: 0.56–0.80; *Z* = −4.52; *p* < 0.001) and GLP‐1RAs versus control had an RR of 0.87 (95% CI: 0.74–1.02; *Z* = −1.69; *p* = 0.091) (Figure 2, panel 2.4).

**FIGURE 3:**
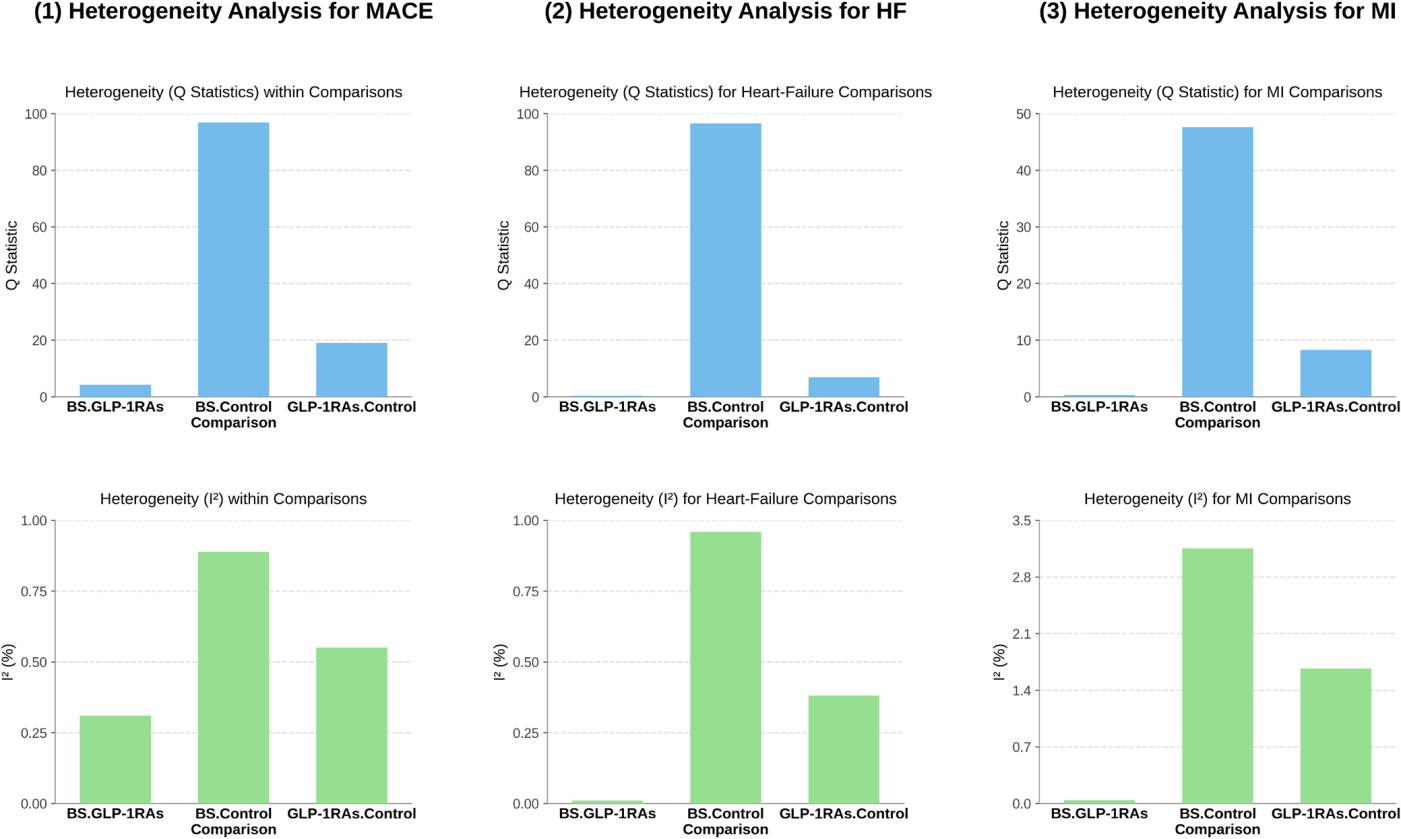
Heterogeneity analysis for hazard ratios (HRs) showing Q statistics and *I* ^2^ values for each treatment comparison. Panel 3.1: Major adverse cardiovascular events (MACEs). Panel 3.2: Heart failure (HF) events. Panel 3.3: Myocardial infarction (MI).

For HRs, heterogeneity was Tau^2^ = 0.02, Tau = 0.15 and *I* ^2^ = 77% (95% CI: 66.1%–84.4%), with Q = 100.04; d.f. = 23; *p* < 0.001. For RRs, heterogeneity was Tau^2^ = 0.08, Tau = 0.28 and *I* ^2^ = 92.5% (95% CI: 89.9%–94.4%) (Figure 3, panel 3.1).

### 3.3 Comparison of HF Events

BS demonstrated the most substantial reduction in HF events, achieving a 52%–55% risk reduction across models. A total of 29 studies provided data on HRs for HF events, with 29 pairwise comparisons. In the fixed‐effects model, BS versus control showed an HR of 0.48 (95% CI: 0.46–0.51; *Z* = −31.97; *p* < 0.001) and GLP‐1RAs versus control had an HR of 0.89 (95% CI: 0.83–0.96; *Z* = −2.94; *p* = 0.003). In the random‐effects model, the HR for BS versus control was 0.45 (95% CI: 0.38–0.53; *Z* = −9.45; *p* < 0.001), whereas GLP‐1RAs versus control showed a non‐significant reduction (HR 0.87; 95% CI: 0.73–1.05; *Z* = −1.46; *p* = 0.145) (Figure 2, panel 2.2).

For RRs, 22 studies assessed the number of HF events, with 22 pairwise comparisons. In the fixed‐effects model, BS versus control had an RR of 0.43 (95% CI: 0.39–0.47; *Z* = −19.19; *p* < 0.001), while GLP‐1RAs versus control had an RR of 0.93 (95% CI: 0.86–1.00; *Z* = −1.94; *p* = 0.052). In the random‐effects model, BS versus control had an RR of 0.40 (95% CI: 0.29–0.56; *Z* = −5.29; *p* < 0.001) and GLP‐1RAs versus control showed a non‐significant RR of 0.84 (95% CI: 0.63–1.14; *Z* = −1.12; *p* = 0.262) (Figure 2, panel 2.5).

For HRs, heterogeneity was Tau^2^ = 0.07, Tau = 0.26 and *I* ^2^ = 79.9% (95% CI: 71.6%–85.7%). For RRs, heterogeneity was Tau^2^ = 0.25, Tau = 0.50 and *I* ^2^ = 92.2% (95% CI: 89.3%–94.2%) (Figure 3, panel 3.2).

### 3.4 Comparison of MIs

BS was associated with a significant reduction in MI risk across all models, while GLP‐1RAs showed significant effects only in fixed‐effects analyses. Data on HRs for MIs were obtained from 25 studies with 25 pairwise comparisons. In the fixed‐effects model, BS versus control had an HR of 0.53 (95% CI: 0.50–0.56; *Z* = −24.18; *p* < 0.001) and GLP‐1RAs versus control had an HR of 0.90 (95% CI: 0.84–0.97; *Z* = −2.88; *p* = 0.004). In the random‐effects model, BS versus control had an HR of 0.53 (95% CI: 0.45–0.63; *Z* = −7.43; *p* < 0.001), whereas GLP‐1RAs versus control showed a non‐significant reduction (HR 0.91; 95% CI: 0.77–1.08; *Z* = −1.04; *p* = 0.30) (Figure 2, panel 2.3).

For RRs, 17 studies assessed MI event counts, with 17 pairwise comparisons. In the fixed‐effects model, BS versus control had an RR of 0.38 (95% CI: 0.33–0.44; *Z* = −12.83; *p* < 0.001) and GLP‐1RAs versus control had an RR of 0.92 (95% CI: 0.86–0.98; *Z* = −2.62; *p* = 0.009). In the random‐effects model, BS versus control had an RR of 0.42 (95% CI: 0.34–0.53; *Z* = −7.65; *p* < 0.001), while GLP‐1RAs versus control showed a non‐significant RR of 0.92 (95% CI: 0.79–1.07; *Z* = −1.08; *p* = 0.280) (Figure 2, panel 2.6).

For HRs, heterogeneity was Tau^2^ = 0.05, Tau = 0.22 and *I* ^2^ = 77% (95% CI: 66.2%–84.4%). For RRs, heterogeneity was Tau^2^ = 0.03, Tau = 0.19 and *I* ^2^ = 66.7% (95% CI: 43.7%–80.3%) (Figure 3, panel 3.3).

### 3.5 Node‐Splitting Analysis for Consistency Assessment

**FIGURE 4:**
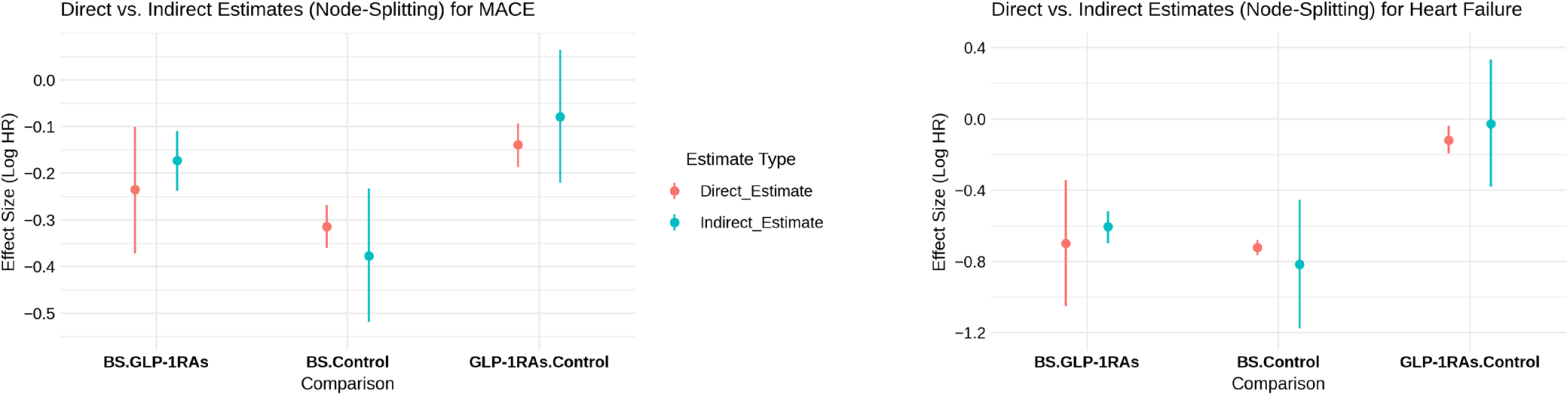
Node‐splitting analysis comparing direct (red) and indirect (blue) hazard ratio (HR) estimates for major adverse cardiovascular events (MACEs) (left) and heart failure (HF) events (right) across treatment comparisons. Effect sizes are presented on a logarithmic scale.

Node‐splitting analysis revealed strong consistency between direct and indirect comparisons across treatment groups for both MACEs and HF events (Figure 4). Node‐splitting analysis for MI was not performed due to the absence of direct comparative evidence for this outcome.

For HRs of MACEs, direct and indirect estimates were closely aligned in both common‐effects and random‐effects models. In the common‐effects model, the NMA estimate for BS versus GLP‐1RAs was 0.54, with direct evidence at 0.50 and indirect evidence at 0.56 (RoR = 0.91; *Z* = −0.50; *p* = 0.617). In the random‐effects model, the NMA estimate was 0.51, with direct evidence at 0.50 and indirect evidence at 0.52 (RoR = 0.98; *Z* = −0.08; *p* = 0.936), with no significant divergence between direct and indirect evidence across any comparison arm.

For HRs of HF events, consistency was similarly maintained. In the common‐effects model, the NMA estimate for BS versus GLP‐1RAs was 0.83, with direct evidence at 0.79 and indirect evidence at 0.84 (RoR = 0.94; *Z* = −0.81; *p* = 0.419). In the random‐effects model, the NMA estimate was 0.78, with direct evidence at 0.82 and indirect evidence at 0.76 (RoR = 1.07; *Z* = 0.52; *p* = 0.600). Across all comparisons, *p*‐values for inconsistency ranged from 0.419 to 0.936, indicating no statistically significant inconsistency between direct and indirect evidence. These results support the robustness of the network model.

### 3.6 Funnel Plots for Publication Bias Assessment

**FIGURE 5:**
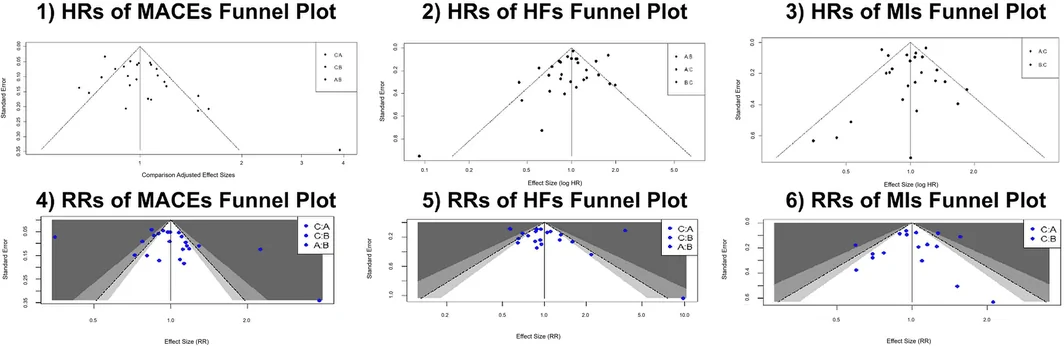
Funnel Plots for Publication Bias Assessment. The plot shows data points for each study with standard errors plotted against effect sizes.

Funnel plots were constructed to assess the presence of publication bias in the comparison‐adjusted effect sizes for HRs and RRs of MACEs, HF events and MIs across the treatment groups. Visual inspection suggests a relatively symmetrical distribution in most plots, indicating a low likelihood of publication bias (Figure 5).

## 4 Discussion

Across 43 studies involving 932,380 patients, the analyses on random‐effects models show that BS significantly reduced the risk of MACEs by approximately 34% (HR 0.66), HF by 55% (HR 0.45) and MI by 47% (HR 0.53). GLP‐1RAs also reduced MACEs by 15% (HR 0.85) yet showed greater variability and non‐significant effects on HF and MI. The fixed‐effects models corroborated these findings with smaller effect sizes as BS reduced the risk of MACEs by 27.4%, while GLP‐1RAs reduced it by 12.6%. These results suggest a meaningful hierarchy of CV efficacy between the two interventions with superior effects of BS across all examined CV outcomes, which was consistent with a 29% lower risk of MACE compared to GLP‐1RAs in another meta‐analysis [62].

BS led to a significant 50%–60% reduction in HF events, varying by the model used. In contrast, GLP‐1RAs showed an 11% reduction in the fixed‐effects HR model, which was non‐significant in random‐effects analyses. A primary limitation is the fundamental difference in study design between the intervention groups. Most evidence for BS is derived from observational cohorts, whereas GLP‐1RA data is largely from RCTs, which may introduce residual confounding in the surgical arm. While this heterogeneity warrants cautious interpretation, the NMA framework serves as the best available method to synthesize such diverse evidence when head‐to‐head RCTs are limited. Other factors contributing to this variability include differences in intervention protocols (e.g., type of BS or GLP‐1RA), inconsistencies in outcome definitions and variable baseline patient characteristics. These findings highlight that while BS appears to have a more consistent effect on HF prevention, the comparative efficacy of GLP‐1RAs requires further investigation in head‐to‐head trials.

BS was associated with reductions of the risk of MI, ranging from 47% (HRs) to 62% (RRs) across models. GLP‐1RAs showed a modest 10% reduction in fixed‐effects HR analyses, which did not reach significance in random‐effects models. Moderate to high heterogeneity was observed across MI analyses. The absence of direct evidence for comparing BS versus GLP‐1RA for MI limited the precision of indirect estimates for this outcome and should be considered when interpreting these results. While BS demonstrates consistent benefits in MI prevention, further research is needed to compare with the efficacy of GLP‐1RAs in this context.

High heterogeneity across both HR‐ and RR‐based analyses warrants careful consideration when interpreting these findings. It reflects considerable variation in follow‐up durations and study designs, particularly between GLP‐1 RCTs and observational BS cohorts. Several other factors may also contribute to this variability, including intervention heterogeneity (multiple GLP‐1RAs and distinct bariatric procedures) with potentially diverse impacts or mechanisms and differences in baseline patient characteristics (e.g., prevalence of type 2 diabetes or severity of obesity). Formal subgroup analyses and meta‐regression were not performed due to data limitations. Future analyses incorporating these approaches would help further elucidate sources of heterogeneity. For instance, studies comparing the CV impact of newer GLP‐1RAs (e.g., tirzepatide) with earlier generations, or distinct BS methods (e.g., Sleeve gastrectomy and Roux‐en‐Y gastric bypass) are warrented.

The node‐splitting analysis revealed strong consistency between direct and indirect comparisons for HRs of MACEs and HF events across treatment groups. For MACEs, direct and indirect estimates were closely aligned in both the common‐effects and random‐effects models, with no significant differences observed (RoR *p*‐values ranging from 0.62 to 0.94). Similarly, for HF events, the estimates showed no significant inconsistency between direct and indirect evidence (RoR *p*‐values ranging from 0.42 to 0.60). These results demonstrate the internal validity of the NMA, indicating that indirect comparisons effectively complement the limited direct evidence, even in scenarios with moderate to high heterogeneity. The consistency across models and treatment groups underscores the reliability of the findings in this analysis. The comparison for MI was not performed due to the absence of direct evidence, which may limit the robustness and reliability of the results for this outcome.

A growing body of evidence described the weight loss‐dependent and ‐independent mechanisms of the CV benefits of both BS and GLP‐1RAs, specifically improvements in insulin sensitivity, blood pressure and systemic inflammation. In addition, meta‐analytic evidence suggests that BS modifies several atherogenic lipoprotein pathways by enhancing cholesterol efflux capacity of high‐density lipoprotein (HDL) particles, significant reductions in low‐density lipoprotein (LDL) and lipoprotein(a) concentrations [63, 64, 65]. GLP‐1RAs also exert direct and indirect CV mechanisms including desirable effects on natriuresis, vascular, myocardial and endothelial tissues [66]. The broader and more durable metabolic remodelling induced by BS may partly explain the greater CV benefit, whereas GLP‐1RAs induce lesser magnitude of weight loss through sustained pharmacological modulation of metabolic, inflammatory and atherosclerotic pathways.

This study's methodological strengths enhance the reliability and applicability of its findings. The NMA allowed for synthesizing data from multiple high‐quality studies, enabling comprehensive comparisons between BS and GLP‐1RAs. This approach provides a robust framework for evaluating interventions where direct comparisons are limited and ensures that conclusions are grounded in a broad evidence base [67, 68]. Including diverse studies would offer a detailed understanding of how each intervention impacts CV outcomes.

Certain limitations must be acknowledged. NMA inherently rely on indirect comparisons, which can introduce potential biases due to study population and design variations. The parallel use of HRs and RRs reflects heterogeneity in outcome reporting across included studies rather than a methodological preference. Although, the directional consistency across both strengthens reliability in the overall conclusions. Additionally, the included studies' heterogeneity could not be formally explored through subgroup analyses or meta‐regression due to data limitations, which may influence the findings' generalizability to all patient populations. Moreover, the predominance of observational studies in the BS arm introduces the possibility of residual confounding, as the patient characteristics may differ among the two methods. The scarecity of large‐scale BS versus GLP‐1RAs comparing RCTs represent methodological constraints. The exisiting RCTs comprise of a combination of mostly first generation GLP‐1RAs (Table S1). Therefore, future research should focus on investigating subgroup analyses stratified by type of BS or specific (i.e., newer) GLP‐1RA agents, diabetes status, BMI category and baseline CV risks to clarify whether particular patient profiles benefit differently from one approach over the other.

Our findings offer important clinical implications for treatment selection in patients with obesity at high CV risk. BS may be the preferred intervention in patients with obesity who are surgical candidates with high CV risk, particularly those with established HF or prior MI. However, GLP‐1RAs remain an effective alternative to improve MACE risk when surgery is contraindicated. In summary, the choice between interventions should be individualized, accounting for surgical risk, patient preference, comorbidity profile and healthcare access.

## 5 Conclusion

This NMA indicates that BS and GLP‐1RAs significantly reduce the risk of MACEs. Furthermore, BS consistently lowers the CV risk more effectively than GLP‐1RAs across all examined outcomes, with more substantial reductions in HF and MI (i.e., more modest and variable reductions with GLP‐1RAs). These findings support BS as the more effective intervention for CV risk reduction in patients with obesity. Nonetheless, GLP‐1RAs remain a clinical alternative, particularly for patients who are not suitable surgical candidates. The high heterogeneity underscores the need for careful interpretation and further research. While both interventions are effective in reducing CV risks, additional studies are necessary to clarify their comparative efficacy and aid in treatment decision‐making.

## Author Contributions

**Erfan Shirmohammadi:** writing – original draft, writing – review and editing, formal analysis, methodology, conceptualization, project administration, data curation. **Navid Ebrahimi:** writing – review and editing, writing – original draft, data curation, conceptualization. **Amir Ehtemami:** writing – review and editing, conceptualization, data curation, writing – original draft. **Narjes Mohammadzadeh:** writing – review and editing, supervision, conceptualization. **Negar Ghasemloo:** writing – original draft, writing – review and editing, data curation, formal analysis, conceptualization. **Mahan Babaei:** writing – original draft, data curation, conceptualization. **Shakiba Fardoost:** writing – original draft, data curation, conceptualization.

## Funding

The authors have nothing to report.

## Ethics Statement

The authors have nothing to report.

## Conflicts of Interest

The authors declare no conflicts of interest.

## Supporting information


**Table S1:** Characteristics of included studies patient populations.

## Data Availability

The datasets generated and analysed during the current study are available from the corresponding author on reasonable request.
