# Supplementary material for: Comparative Cardiovascular Outcomes of GLP‐1 Receptor Agonists Versus Bariatric Surgery: A Systematic Review and Network Meta‐Analysis
Source: Endocrinol Diabetes Metab. 2026 Sep 5;9(5):e70311. doi: 10.1002/edm2.70311 (PMC13546078; doi:10.1002/edm2.70311)
Supplement: Supplementary file 1 [file EDM2-9-e70311-s001.docx]

**Supplementary table 1.** Characteristics of included studies patient populations

| Author name (year) | Study groups | Drug/surgery type | Study design | No. of cases | No. of Controls | HR MACE | HR MACE (lower CI) | HR MACE (upper CI) | HR MI | HR MI (lower CI) | HR MI (upper CI) | HR HF | HR HF (lower CI) | HR HF (upper CI) | No. MACE case | No. MACE control | No. MI case | No. MI control | No. HF case | No. HF control |
| --- | --- | --- | --- | --- | --- | --- | --- | --- | --- | --- | --- | --- | --- | --- | --- | --- | --- | --- | --- | --- |
| Bouchard et al. 2022 | Bariatric vs. Control | AGB-SG-RYGB-DS | Cohort | 3627 | 5420 | 0.83 | 0.78 | 0.89 |  |  |  | 0.8 | 0.7 | 0.9 | 712.0 | 1346.0 |  |  | 182.0 | 377.0 |
| Alkharaiji et al. 2019 | Bariatric vs. Control | SG-RYGB | Cohort | 131 | 579 |  |  |  | 0.98 | 0.54 | 1.77 | 0.89 | 0.47 | 1.7 |  |  | 13.0 | 95.0 | 13.0 | 91.0 |
| Sampalis et al. 2006 | Bariatric vs. Control | RYGB-VG | Cohort | 1035 | 5746 |  |  |  | 0.71 | 0.5 | 1.002 |  |  |  |  |  | 35.0 | 274.0 |  |  |
| Romeo et al. 2012 | Bariatric vs. Control | RYGB-GB-VG | Cohort | 345 | 262 |  |  |  | 0.56 | 0.34 | 0.93 |  |  |  |  |  |  |  |  |  |
| Douglas et al. 2015 | Bariatric vs. Control | AGB-SG-RYGB | Cohort | 3882 | 3882 |  |  |  | 0.28 | 0.1 | 0.74 |  |  |  |  |  | 5.0 | 18.0 |  |  |
| Eliasson et al. 2015 | Bariatric vs. Control | RYGB | Cohort | 6132 | 6132 |  |  |  | 0.49 | 0.24 | 1.01 |  |  |  |  |  | 15.0 | 39.0 |  |  |
| Benotti et al. 2017 | Bariatric vs. Control | RYGB | Cohort | 1724 | 1724 |  |  |  | 0.89 | 0.41 | 1.92 | 0.38 | 0.22 | 0.64 |  |  | 12.0 | 17.0 | 24.0 | 55.0 |
| Brown et al. 2022 | Bariatric vs. Control | AGB-SG-RYGB | Cohort | 60445 | 268362 |  |  |  | 0.39 | 0.35 | 0.42 |  |  |  |  |  |  |  |  |  |
| Moussa et al . 2020 | Bariatric vs. Control | AGB-SG-RYGB | Cohort | 3701 | 3701 | 0.41 | 0.274 | 0.615 | 0.412 | 0.28 | 0.606 | 0.403 | 0.181 | 0.897 |  |  | 37.0 | 93.0 | 22.0 | 46.0 |
| Stenberg et al. 2020 (I) | Bariatric vs. Control | SG-RYGB | Cohort | 11863 | 26199 | 0.73 | 0.64 | 0.84 | 0.53 | 0.42 | 0.67 |  |  |  | 379.0 | 1125.0 |  |  |  |  |
| Wong et al. 2021 | Bariatric vs. Control | SG-RYGB | Cohort | 303 | 1399 |  |  |  | 0.534 | 0.125 | 2.278 | 0.283 | 0.068 | 1.173 |  |  |  |  |  |  |
| Hoskuldsdottir et al. 2020 | Bariatric vs. Control | RYGB | Cohort | 387 | 387 |  |  |  | 0.57 | 0.24 | 1.35 | 0.32 | 0.15 | 0.67 |  |  |  |  |  |  |
| Dash et al. 2021 | Bariatric vs. Control | SG-RYGB | Cohort | 3098 | 5470 |  |  |  | 0.519 | 0.301 | 0.894 | 0.198 | 0.109 | 0.36 |  |  |  |  |  |  |
| Hung et al. 2021 | Bariatric vs. Control | LSG-GB-OAGB | Cohort | 1436 | 1436 |  |  |  | 0.186 | 0.054 | 0.643 |  |  |  |  |  | 3.0 | 15.0 |  |  |
| Lundberg et al. 2021 | Bariatric vs. Control | RYGB | Cohort | 28204 | 40827 |  |  |  | 0.6 | 0.41 | 0.88 |  |  |  |  |  | 97.0 | 518.0 |  |  |
| Yuan et al. 2021 | Bariatric vs. Control | RYGB | Cohort | 308 | 701 | 0.62 | 0.44 | 0.88 | 0.24 | 0.07 | 0.77 |  |  |  |  |  |  |  |  |  |
| Mentias et al. 2022 | Bariatric vs. Control | SG-GB | Cohort | 94885 | 94885 |  |  |  | 0.63 | 0.59 | 0.68 | 0.46 | 0.44 | 0.49 |  |  |  |  |  |  |
| Aminian et al. 2019 | Bariatric vs. Control | AGB-SG-RYGB-DS | Cohort | 2287 | 39267 | 0.61 | 0.55 | 0.69 |  |  |  | 0.38 | 0.3 | 0.49 | 194.0 | 1765.0 |  |  |  |  |
| Singh et al. 2020 | Bariatric vs. Control | AGB-SG-RYGB-DS | Cohort | 5170 | 9995 |  |  |  |  |  |  | 0.57 | 0.34 | 0.96 |  |  |  |  |  |  |
| Rassen et al. 2021 | Bariatric vs. Control | SG-RYGB | Cohort | 344 | 551 | 0.99 | 0.76 | 1.3 |  |  |  | 0.82 | 0.44 | 1.52 |  |  |  |  |  |  |
| Persson et al. 2017 | Bariatric vs. Control | VG-GB | Cohort | 22295 | 25564 |  |  |  |  |  |  | 0.37 | 0.29 | 0.46 |  |  |  |  | 89.0 | 944.0 |
| Jamaly et al. 2019 | Bariatric vs. Control | AGB-VG-RYGB | Cohort | 2003 | 2030 |  |  |  |  |  |  | 0.65 | 0.54 | 0.79 |  |  |  |  | 188.0 | 266.0 |
| Liakopoulos et al. 2020 | Bariatric vs. Control | GB | Cohort | 5321 | 5321 |  |  |  |  |  |  | 0.33 | 0.24 | 0.46 |  |  |  |  | 86.0 | 233.0 |
| Hoskuldsdottir et al. 2021 | Bariatric vs. Control | RYGB | Cohort | 5321 | 5321 |  |  |  |  |  |  | 0.27 | 0.19 | 0.38 |  |  |  |  | 47.0 | 151.0 |
| Doumouras et al. 2021 | Bariatric vs. Control | SG-GB | Cohort | 1319 | 1319 | 0.58 | 0.48 | 0.7 |  |  |  |  |  |  |  |  |  |  |  |  |
| Naslund et al. 2021 | Bariatric vs. Control | SG-RYGB | Cohort | 509 | 509 | 0.44 | 0.32 | 0.61 |  |  |  |  |  |  |  |  |  |  |  |  |
| Pirlet et al. 2020 | Bariatric vs. Control | SG-GB-DS | Cohort | 116 | 116 | 0.65 | 0.42 | 1.0 |  |  |  |  |  |  |  |  |  |  |  |  |
| Sjostrom, et al. 2012 | Bariatric vs. Control | GB-VG | Cohort | 2010 | 2037 | 0.67 | 0.54 | 0.83 |  |  |  |  |  |  |  |  |  |  |  |  |
| Kosiborod et al. 2024 (I) | GLP-1RA vs. Control | Semaglutide | Cohort | 310 | 306 |  |  |  |  |  |  | 0.4 | 0.15 | 0.92 |  |  |  |  | 7.0 | 18.0 |
| Kosiborod et al. 2023 (II) | GLP-1RA vs. Control | Semaglutide | Cohort | 263 | 266 |  |  |  |  |  |  | 0.08 | 0.01 | 0.42 |  |  |  |  | 1.0 | 12.0 |
| Lincoff et al. 2023 | GLP-1RA vs. Control | Semaglutide | Cohort | 8801 | 8803 | 0.8 | 0.71 | 0.9 | 0.72 | 0.61 | 0.85 | 0.82 | 0.71 | 0.96 | 569.0 | 701.0 | 234.0 | 322.0 | 97.0 | 122.0 |
| Ruff et al. 2022 | GLP-1RA vs. Control | Exenatide | Cohort | 2075 | 2081 | 1.22 | 0.9 | 1.65 | 1.33 | 0.81 | 2.18 | 0.94 | 0.48 | 1.87 | 95.0 | 79.0 | 37.0 | 28.0 | 16.0 | 17.0 |
| Gerstein et al. 2021 | GLP-1RA vs. Control | Efpeglenatide | RCT | 2717 | 1359 | 0.73 | 0.58 | 0.92 | 0.75 | 0.54 | 1.05 | 0.61 | 0.38 | 0.98 | 189.0 | 125.0 | 85.0 | 53.0 | 40.0 | 31.0 |
| Marso et al. 2020 (I) | GLP-1RA vs. Control | Liraglutide | RCT | 4668 | 4672 | 0.87 | 0.78 | 0.97 | 0.88 | 0.75 | 1.03 | 0.87 | 0.73 | 1.05 | 608.0 | 694.0 | 281.0 | 317.0 | 218.0 | 248.0 |
| Gerstein et al. 2019 (I) | GLP-1RA vs. Control | Dulaglutide | RCT | 4949 | 4952 | 0.88 | 0.79 | 0.99 | 0.96 | 0.79 | 1.16 | 0.93 | 0.77 | 1.12 | 594.0 | 663.0 | 205.0 | 212.0 | 213.0 | 226.0 |
| Husain et al. 2019 (II) | GLP-1RA vs. Control | Semaglutide | RCT | 1591 | 1592 | 0.8 | 0.56 | 1.12 | 1.2 | 0.74 | 1.94 | 0.86 | 0.48 | 1.55 | 61.0 | 76.0 | 37.0 | 31.0 | 21.0 | 24.0 |
| Hernandez et al. 2018 | GLP-1RA vs. Control | Albiglutide | RCT | 4731 | 4732 | 0.78 | 0.68 | 0.9 |  |  |  |  |  |  | 338.0 | 428.0 |  |  |  |  |
| Margulies et al. 2016 | GLP-1RA vs. Control | Liraglutide | RCT | 154 | 146 |  |  |  |  |  |  | 1.33 | 0.83 | 2.12 |  |  |  |  | 63.0 | 50.0 |
| Holman et al. 2017 | GLP-1RA vs. Control | Exenatide | RCT | 7356 | 7396 | 0.91 | 0.83 | 1.0 | 0.97 | 0.85 | 1.10 | 0.94 | 0.78 | 1.13 | 839.0 | 905.0 | 455.0 | 470.0 | 219.0 | 231.0 |
| Marso et al. 2016 (II) | GLP-1RA vs. Control | Semaglutide | RCT | 1648 | 1649 | 0.74 | 0.58 | 0.98 | 0.74 | 0.51 | 1.08 | 1.11 | 0.77 | 1.61 | 108.0 | 146.0 | 47.0 | 64.0 | 59.0 | 54.0 |
| Pfeffer et al. 2015 | GLP-1RA vs. Control | Lixisenatide | RCT | 3034 | 3034 | 1.02 | 0.88 | 1.18 | 1.04 | 0.86 | 1.24 | 0.96 | 0.74 | 1.24 | 406.0 | 399.0 | 255.0 | 247.0 | 122.0 | 127.0 |
| Dicker et al. 2024 (I) | GLP-1RA vs. Bariatric | First generation (liraglutide, dulaglutide, exenatide, lixisenatide) | RCT | 2371 | 2371 | 0.74 | 0.49 | 1.1 |  |  |  |  |  |  | 69.0 | 109.0 |  |  |  |  |
| Stenberg et al. 2024 (II) | GLP-1RA vs. Bariatric | liraglutide, dulaglutide, or semaglutide | RCT | 2039 | 2039 | 0.75 | 0.62 | 0.91 |  |  |  |  |  |  | 191.0 | 247.0 |  |  |  |  |
| Stenberg et al. 2023 (III) | GLP-1RA vs. Bariatric | liraglutide, dulaglutide, exenatide, lixisenatide and semaglutide | RCT | 2161 | 2161 | 0.76 | 0.59 | 0.98 |  |  |  | 0.61 | 0.35 | 1.05 | 113.0 | 130.0 |  |  | 24.0 | 34.0 |
| Wolff Sagy et al. 2024 | GLP-1RA vs. Bariatric | liraglutide, exenatide, lixisenatide, dulaglutide and semaglutide | RCT | 2205 | 2205 |  |  |  |  |  |  | 0.43 | 0.27 | 0.68 |  |  |  |  | 26.0 | 90.0 |

AGB: adjustable gastric band; DS: duodenal switch; GLP-1RA: glucagon-like peptide-1 receptor agonist; HF: heart failure; MACE: major adverse cardiovascular events; MI: myocardial infarction OAGB: one anastomosis gastric bypass; RYGB: Roux-en-Y gastric bypass; SG: sleeve gastrectomy; vertical gastrectomy
